# The capillary lobule variant of radiation‐associated angiosarcoma in the setting of breast cancer: A diagnostic pitfall

**DOI:** 10.1111/cup.14328

**Published:** 2022-10-10

**Authors:** Shruti Agrawal, Karen J. Fritchie, Anthony P. Fernandez, Jennifer S. Ko, Wilma Bergfeld, Brian P. Rubin, Steven D. Billings

**Affiliations:** ^1^ Department of Pathology Cleveland Clinic Cleveland Ohio USA; ^2^ Department of Dermatology Cleveland Clinic Cleveland Ohio USA

**Keywords:** angiosarcoma, breast cancer, capillary lobule, immunohistochemistry, radiation

## Abstract

**Aim:**

Post‐radiation angiosarcoma is an iatrogenic event seen in the setting of breast cancer treatment. Histopathologically, there are morphologic variants of angiosarcoma that mimic benign entities, including the capillary lobule variant of post‐radiation angiosarcoma. We present the largest case series to date of this histopathologic variant of post‐radiation angiosarcoma.

**Methods and Results:**

Cases of the capillary lobule variant of post‐radiation angiosarcoma from institutional/consultation archives from 2008 to June 2022 were reviewed. For inclusion, tumors had to occur in irradiated skin and exhibit a multi‐lobular proliferation of tightly packed capillary‐like vessels, as previously described in this variant. Prior ancillary studies were also reviewed. Eight cases met the criteria. All occurred in women treated with radiation for breast cancer (median age 75 years). All cases had similar findings, including a multi‐lobular proliferation of tightly packed vessels, infiltrative cords, and atypical single endothelial cells. A conventional angiosarcoma pattern was also seen in five cases. All cases tested were positive for vascular markers (CD31, CD34, and/or ERG) and MYC. *MYC* amplification was shown by FISH in all cases tested. Smooth muscle actin (SMA) was positive in pericytes in the capillary lobules in all five cases tested and areas of conventional angiosarcoma in two of three cases.

**Conclusions:**

The capillary lobule variant of angiosarcoma is a rare and therefore potentially under‐recognized variant of post‐radiation angiosarcoma. The lobular architecture and SMA positivity may mimic benign vascular proliferations. Careful attention to histopathologic features and ancillary tests may facilitate accurate diagnosis.

## 1 INTRODUCTION

Post‐radiation angiosarcoma is a rare iatrogenic event most commonly seen in the setting of breast cancer treatment.^1^, ^2^ Histopathologically, the morphologic appearance is usually that of conventional angiosarcoma, which can vary from dissecting vascular channels, a sieve‐like pattern, a spindled morphology, an epithelioid morphology, to solid sheets of atypical endothelial cells.^1^, ^2^ Individual tumors may have multiple patterns in the same neoplasm. Cytologic atypia, multi‐layering, and mitotic activity are frequently noted.^1^, ^2^ A subtle histopathological variant of angiosarcoma mimicking radiation dermatitis has also been described recently.^3^ To the best of our knowledge, there are only two prior papers in the literature detailing the capillary lobule variant of post‐radiation angiosarcoma.^4^, ^5^ In the original study, three cases of post‐radiation angiosarcoma characterized by circumscribed lobules of neoplastic vessels were described.^4^ It was noted that this histopathologic pattern could be confused with benign vascular tumors.^4^, ^5^ Herein, we report the largest case series to date of the capillary lobule pattern of post‐radiation angiosarcoma, a potentially under‐recognized and diagnostically challenging morphologic variant of post‐radiation angiosarcoma.

## 2 MATERIALS AND METHODS

Following Institutional Review Board approval (IRB #07‐576), hospital and consultation pathology archives from the senior author's institution were searched for cases of the capillary lobule variant of post‐radiation angiosarcoma of the breast from 2008 to June 2022. Required inclusion criteria included histopathologic features that were consistent with this variant of angiosarcoma, in addition to an associated history of radiation to the affected area. Available H&E‐stained slides, immunohistochemistry slides, and scanned digital images were reviewed. Results of prior fluorescence in situ hybridization (FISH) for *MYC* amplification were documented.

## 3 RESULTS

**FIGURE 1:**
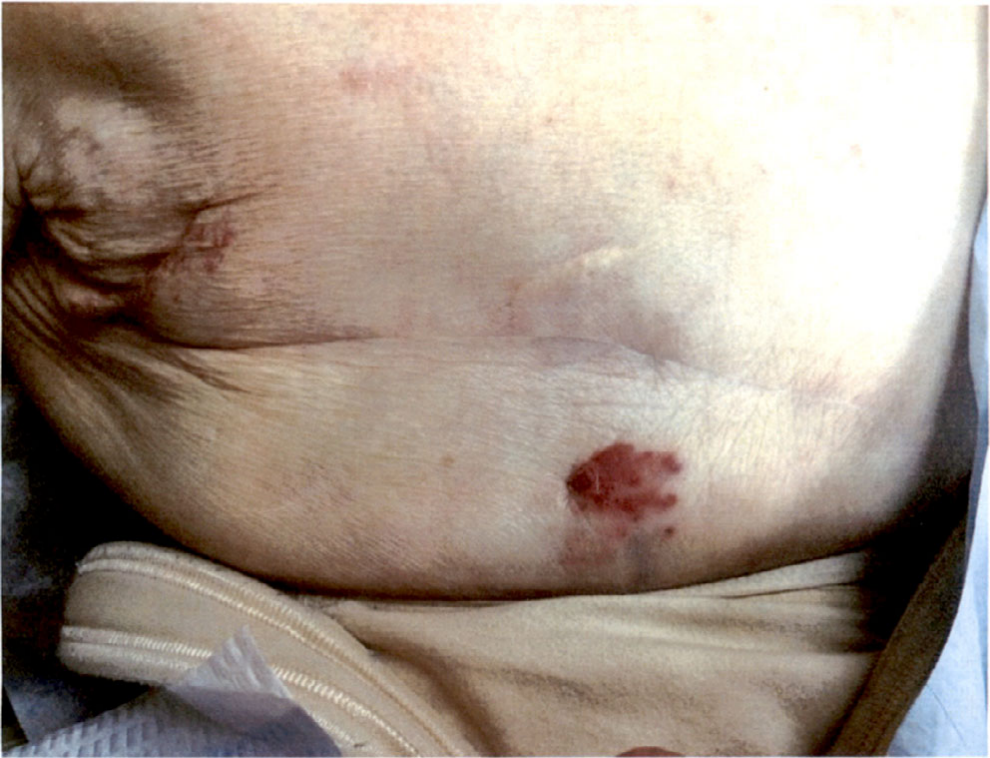
An approximately 2‐cm red, vascular‐appearing plaque on the breast, with a clinical differential diagnosis of basal cell carcinoma, squamous cell carcinoma, breast cancer, and angiosarcoma

A total of eight cases met the selection criteria. The cases were exclusively from the breast of women who underwent radiation for breast cancer, with a median age of 75 years at the time of biopsy (range, 55–96 years). Clinical information and follow‐up data were limited, as the majority of cases were reviewed in consultation. The patients presented between 4 and 13 years after radiation therapy was performed (median latency of 5 years). One patient had a prior history of angiosarcoma. When descriptions were available, the lesions were clinically described as a “rubbery, roughened cutaneous surface”, “skin thickening with mild erythema” a “smooth‐red violaceous plaque,” “vascular changes on both sides of the areola” with clinical concern for lymphangioma circumscriptum, and a “two centimeter pink plaque” with a clinical differential diagnosis of neoplasm of uncertain behavior, basal cell carcinoma, squamous cell carcinoma, breast cancer, and angiosarcoma (Figure 1). Originating pathologists' diagnoses included: angiosarcoma, post‐radiation atypical vascular lesion, reactive angioendotheliomatosis, reactive vascular proliferation, tufted angioma, glomeruloid hemangioma, and diffuse dermal angiomatosis.

**FIGURE 2:**
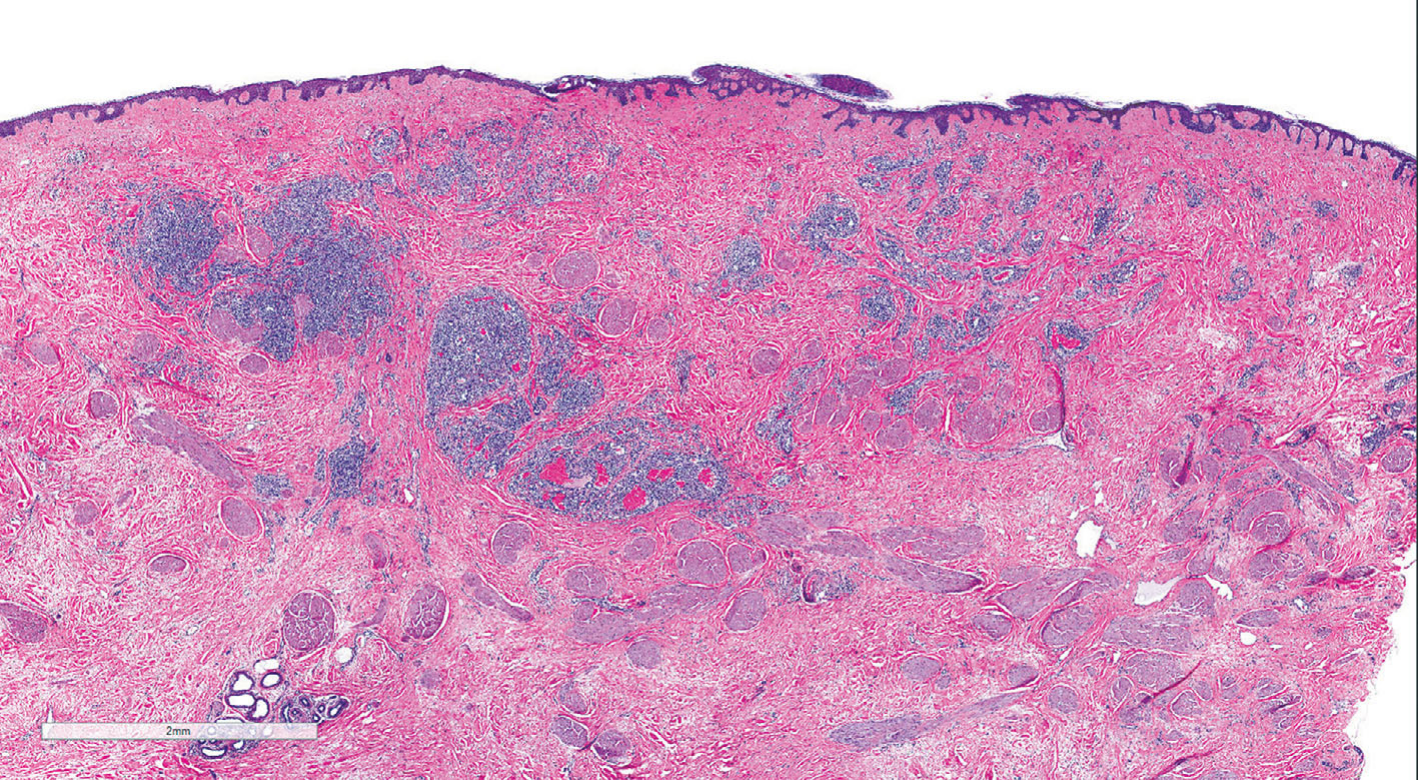
Multiple well‐demarcated lobules of tightly packed capillary‐like vessels scattered throughout the dermis, characteristic of the capillary‐lobule variant of post‐radiation angiosarcoma (H&E, ×15)

**FIGURE 3:**
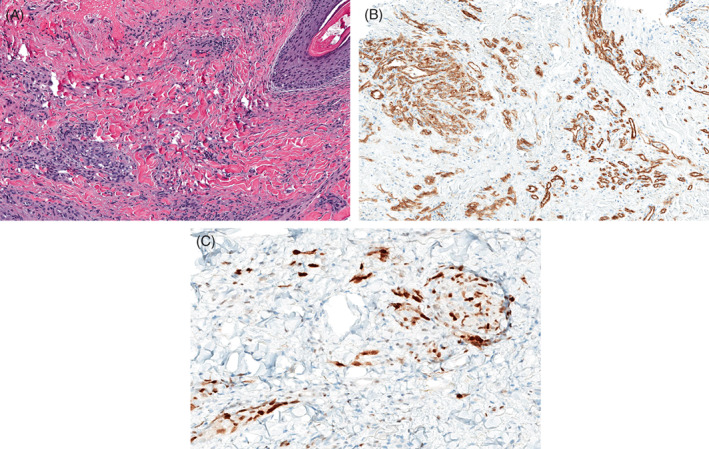
In this case, the capillary lobules were associated with neoplastic vessels that were well‐formed without architectural complexity (A: H&E, ×100). The CD31 stain helps illustrate the architectural features of the capillary lobule and well‐formed neoplastic vessels (B: CD31, ×100). The capillary lobules as well as the well‐formed vessels were positive for MYC (C: MYC, ×200).

**FIGURE 4:**
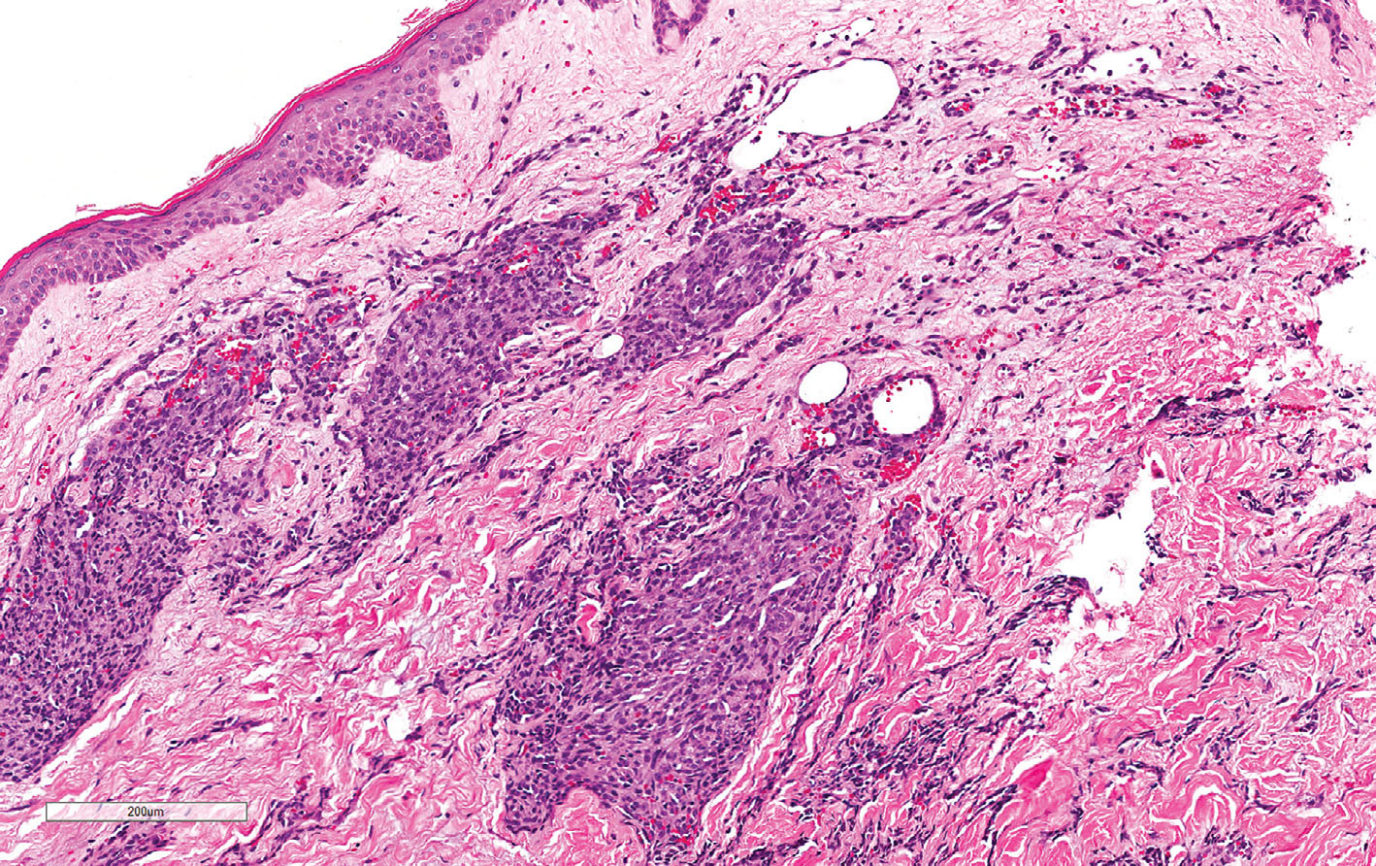
A well‐demarcated capillary lobule is seen, in addition to infiltrative cords and atypical single endothelial cells intercalating through collagen bundles with associated extravasated erythrocytes (H&E, ×100).

**FIGURE 5:**
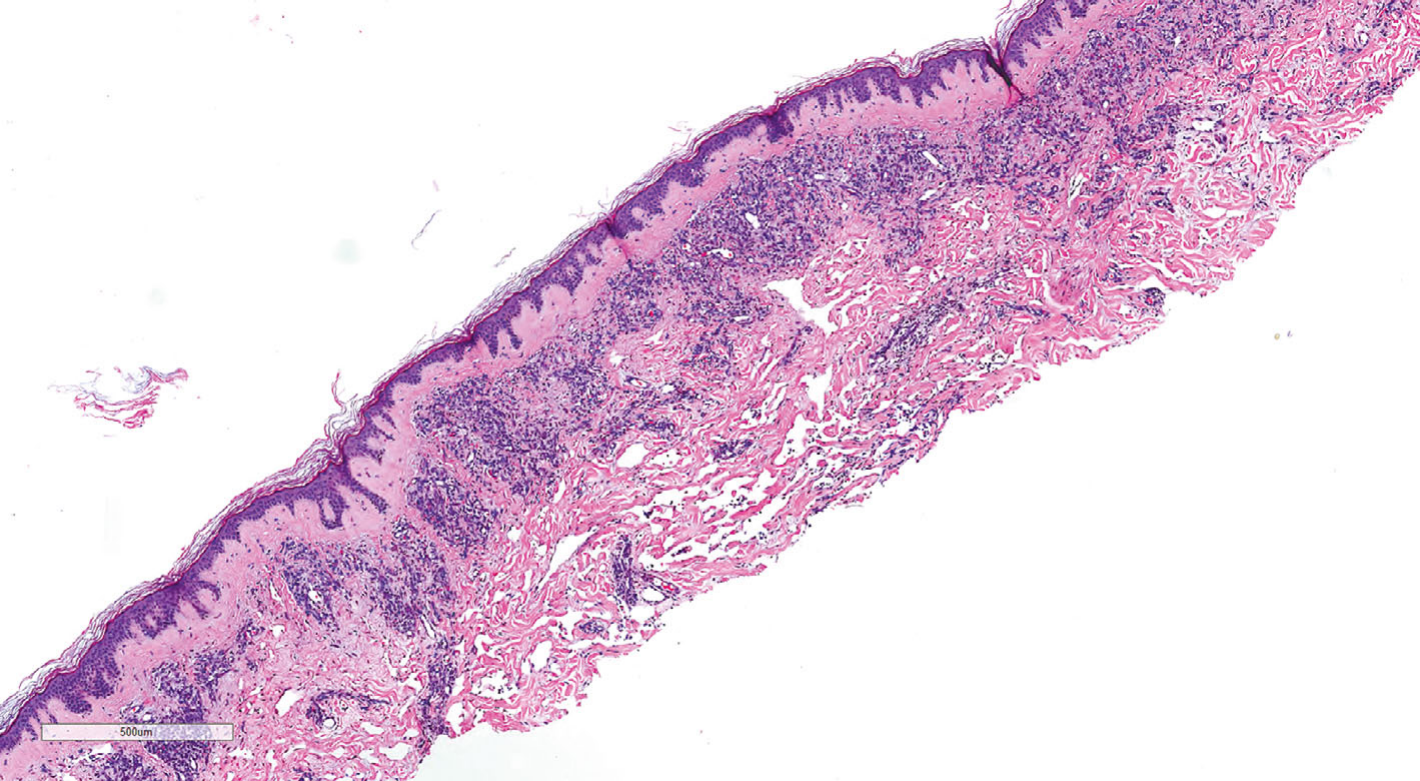
Tightly packed capillary lobules are present in the superficial dermis, with concurrent dilated, architecturally complex anastomosing vessels, reminiscent of conventional angiosarcoma, at the base of the biopsy specimen (H&E, ×40).

**FIGURE 6:**
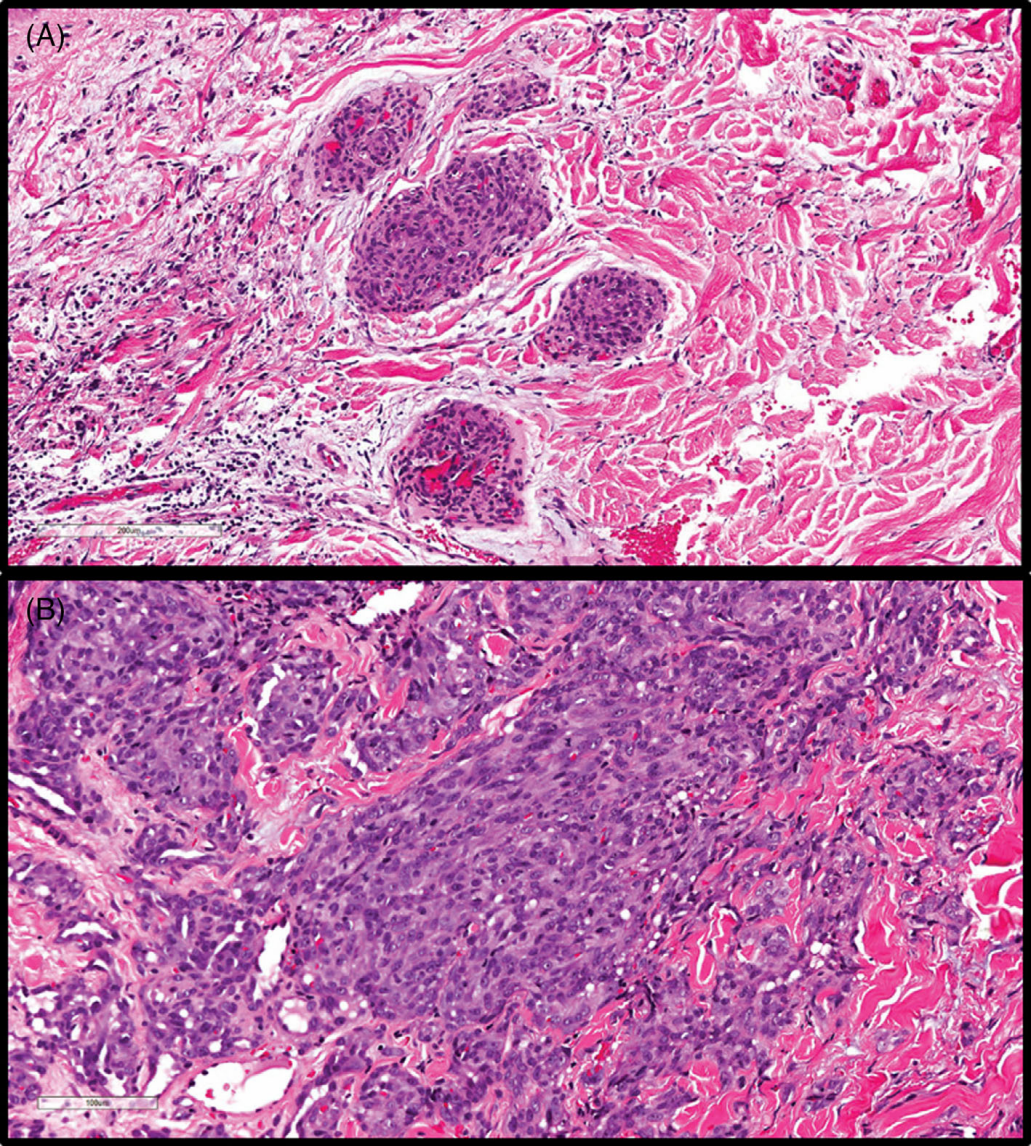
Varying degrees of cytological atypia ranging from mild nuclear enlargement and hyperchromasia (A: H&E, ×100) to more atypical cells (B: H&E, ×200) were noted.

**FIGURE 7:**
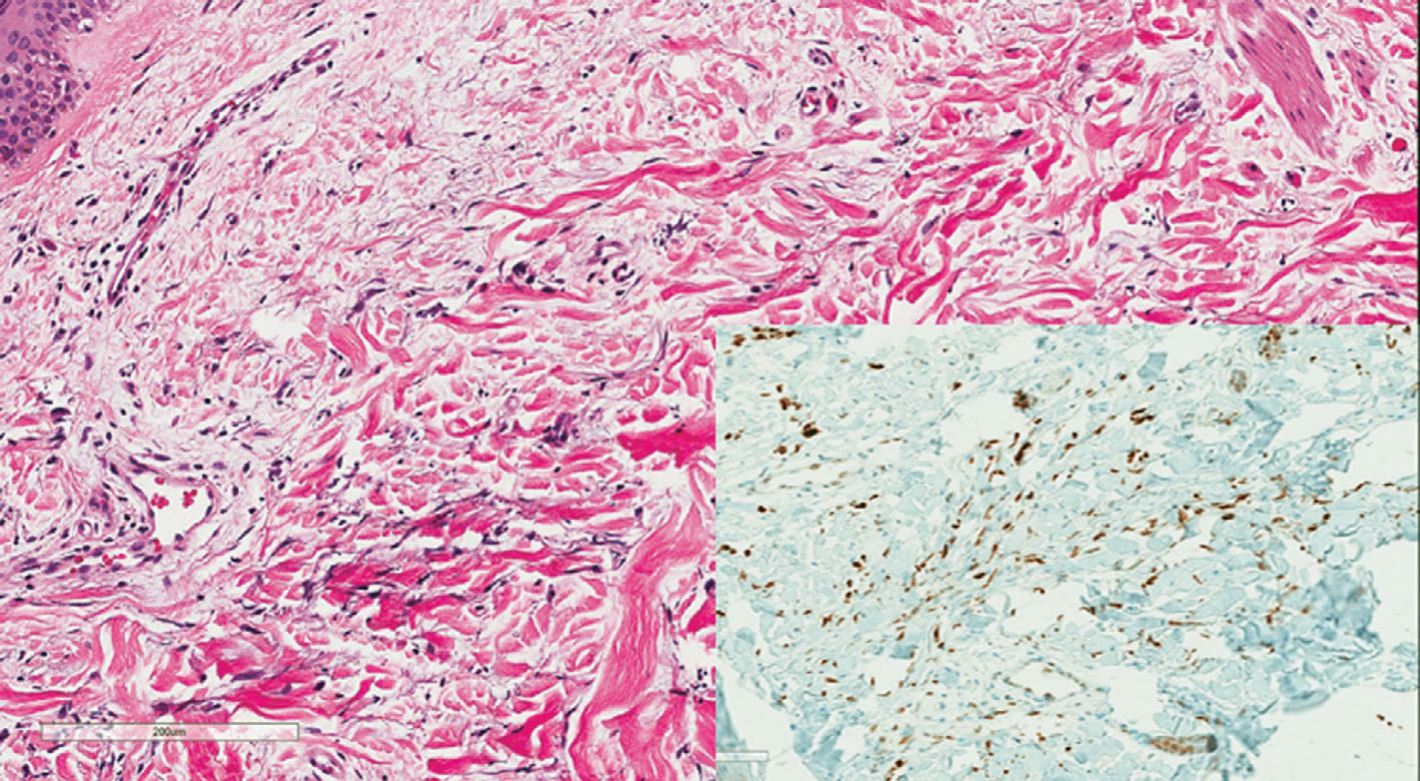
Features of the “radiation‐dermatitis” pattern of angiosarcoma were seen in two cases, with scattered atypical endothelial cells and worm‐like vascular channels (H&E, ×100) that were also MYC positive by immunohistochemistry (inset, ×200).

Histopathologically, all cases showed a multi‐nodular proliferation of tightly packed endothelial cells scattered throughout the dermis (Figure 2). In three cases, there were capillary lobules as well as neoplastic vessels that lacked architectural complexity (Figure 3). The lobules were composed of variably sized but well‐formed vascular lumens lined by plump endothelial cells. In addition, all cases had an infiltrative cord‐like growth pattern and scattered atypical endothelial cells intercalating among the collagen bundles with associated hemorrhage (Figure 4). Areas of more conventional angiosarcoma were seen in the majority of cases (*n* = 5), composed of architecturally complex anastomosing vessels lined by atypical endothelial cells (Figure 5). Overall, the conventional angiosarcoma component constituted the minority of the tumor in the biopsy specimens, but this may not be representative of the entire tumor, given that this study is based on relatively small biopsy samples. All of the cases showed some degree of cytologic atypia, with varying amounts of nuclear enlargement and hyperchromasia of endothelial cells (Figure 6A,B). Mitotic activity was present in seven of eight cases, ranging from 1 to 14 mitotic figures/10 high‐power fields (HPFs) (median 3 mitotic figures/10 HPFs). Concurrent features of the “radiation‐dermatitis”‐like pattern, with scattered atypical single endothelial cells and “worm‐like” vascular channels were also seen in two cases (Figure 7).

**FIGURE 8:**
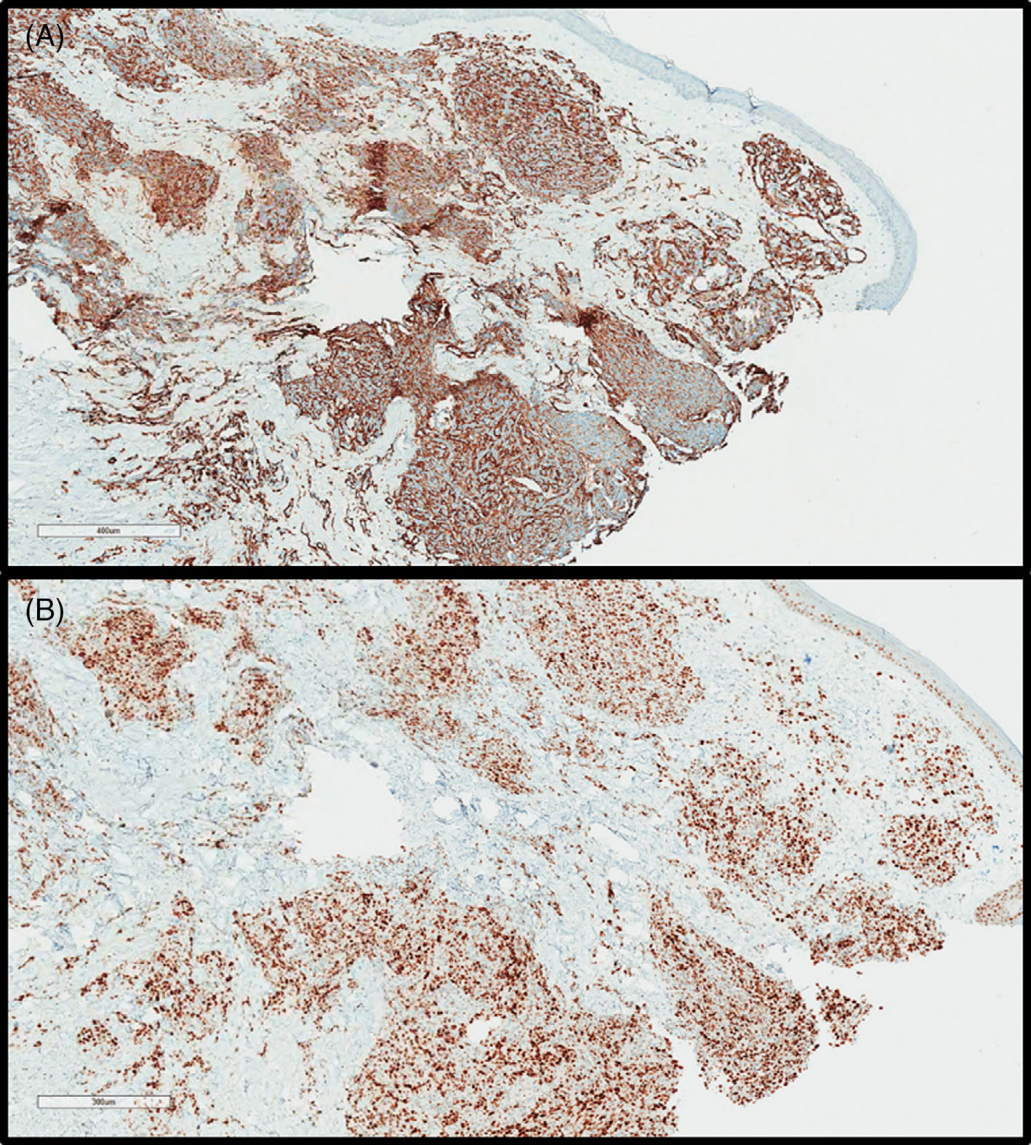
The SMA stain highlights pericytes in capillary lobules, and in infiltrative cords and single cells (A, ×15); the MYC stain is positive in the same lesional cells (B, ×20)

The atypical endothelial cells were positive for vascular markers, including CD31 (6/6), CD34 (4/4), and ERG (1/1). Smooth muscle actin (SMA) was positive in pericytes in the capillary lobules in 5 of 5 cases and areas of conventional angiosarcoma in 2 of 3 cases with overlapping morphologies (Figure 8A). Immunohistochemical stains for MYC showed strong nuclear staining in the atypical endothelial cells of both the capillary lobule component and other patterns of neoplastic vessels in all cases tested (7/7) (Figure 8B). FISH showed *MYC* amplification in all cases tested (3/3).

## 4 DISCUSSION

Here we describe the largest case series to date illustrating the capillary lobule variant of post‐radiation angiosarcoma. Clinically, this variant falls into the spectrum of what has been previously described in post‐radiation angiosarcoma, with a latency shorter than typical post‐radiation angiosarcoma. We are currently unaware of this histopathologic pattern being described in other forms of angiosarcoma, but this may be because of under‐recognition.

While these are typically recognized as vascular lesions on examination, the clinical and histopathologic differential diagnoses are often broad, including both benign and malignant endothelial lesions. To the best of our knowledge, only two papers have been published previously regarding this variant,^4^, ^5^ and given its deceptive morphologic features, we suspect that this histopathologic variant may be under‐recognized. Clinical information was somewhat limited, but a vascular lesion was frequently suspected, although two cases were clinically described as only having “a roughened surface” or “skin thickening with mild erythema,” respectively. For cases sent in consultation, the differential diagnosis from contributing pathologists included angiosarcoma, post‐radiation atypical vascular lesion, reactive angioendotheliomatosis, reactive vascular proliferation, tufted angioma, glomeruloid hemangioma, and diffuse dermal angiomatosis, highlighting the broad overlap of this histopathologic pattern with benign entities.

Histopathologically, these lesions are often vaguely biphasic. The predominant low‐power morphologic features are characterized by a multi‐nodular proliferation of tightly packed neoplastic vessels with variably atypical endothelial cells. Closer inspection also revealed other patterns of angiosarcoma. Conventional angiosarcoma was present in five of eight cases, and the radiation‐dermatitis‐like pattern was seen in two of eight cases, further highlighting the fact that multiple histopathologic patterns of angiosarcoma may be present in a single neoplasm. In two cases, some of the background neoplastic vessels associated with the capillary lobules were deceptively bland and lacked architectural complexity. Importantly, areas of conventional angiosarcoma were often seen at the periphery or base of the biopsy specimen, and constituted a minority of the tumor in these specimens. Therefore, this finding may be missed or overlooked on superficial biopsy specimens. The resection specimens were not available for examination, and we cannot therefore comment on the true proportion of conventional angiosarcoma versus the capillary lobule pattern present in the entire neoplasm.

The capillary‐lobule‐like architecture is notable in that this architectural pattern could potentially be mistaken for a benign vascular tumor, such as tufted angioma or pyogenic granuloma.^6^ Capillary lobules have also been associated with reactive conditions such as venous stasis, acroangiodermatitis, reactive angioendotheliomatosis proliferans, verruga peruana, and bacillary angiomatosis.^6^ Additionally, the presence of SMA‐positive pericytes may cause diagnostic confusion, as it is often thought that angiosarcomas lack pericytes around the neoplastic vessels. Although we are not aware of a definitive study where this has been rigorously investigated, major textbooks highlight the absence of pericytes as a useful feature in the diagnosis of angiosarcoma.^7^

Of those aforementioned entities, acquired tufted angioma, intravascular pyogenic granuloma, and angioendotheliomatosis are the closest histopathologic simulants. Tufted angioma is often described as having a “cannonball” appearance, characterized by vascular nodules scattered in the dermis.^7^ Although there are architectural similarities, tufted angioma usually presents in children to young adults, as opposed to older women with radiation history, as described in this series. Additionally tufted angioma lacks cytologic atypia.^8^, ^9^

Intravascular pyogenic granulomas are characterized by a lobular proliferation of tightly packed capillaries with a central ectatic vessel. However, they frequently have evidence of pre‐existing thick‐walled vessels at the periphery, a feature not seen in any of our cases.^10^ Although intravascular pyogenic granulomas can occasionally have mild cytologic atypia and mitotic activity, prominent cytologic atypia is not a feature.^10^, ^11^ The most helpful feature in differentiating pyogenic granuloma and tufted angioma from the capillary lobule variant of angiosarcoma is the absence of an infiltrative component.

Reactive angioendotheliomatosis generally presents as erythematous macules or purpuric plaques on the extremities.^12^, ^13^ This is generally thought to be a reactive process secondary to ischemia, leading to an intra‐ or extra‐vascular lobular or diffuse proliferation of endothelial cells and pericytes^12^, ^13^ A variant of this condition, acroangiodermatitis, occurs secondary to stasis dermatitis, and is noted to also have a lobular architecture.^14^ Another subtype, “diffuse dermal angiomatosis,” may present in the setting of large pendulous breasts. Despite the similarity in location, this entity typically has a more diffuse and interstitial vascular growth pattern rather than the distinctly nodular architecture seen in our cases.^13^ Cytologic atypia is also not a feature.^13^

To further distinguish between clinical and pathologic mimics, demonstration of *MYC* amplification by FISH analysis or positive MYC expression by immunohistochemistry can support a diagnosis of post‐radiation angiosarcoma.^15^, ^16^

In summary, we present a series of a rare morphologic variant of post‐radiation angiosarcoma termed the capillary lobule pattern. Features that allow accurate diagnosis include the clinical presentation in previously irradiated breast skin, the presence of cytologic atypia, areas resembling radiation‐dermatitis‐like angiosarcoma, infiltrative areas resembling conventional angiosarcoma, and demonstration of expression of MYC by immunohistochemistry or *MYC* amplification by FISH. Careful attention to these clinical and histopathologic findings should prevent misdiagnosis as a benign vascular tumor.

## CONFLICT OF INTEREST

The authors declare no conflict of interest.
